# *Fonsecaea pedrosoi*‐induced Th17‐cell differentiation in mice is fostered by Dectin‐2 and suppressed by Mincle recognition

**DOI:** 10.1002/eji.201545591

**Published:** 2015-07-28

**Authors:** Marcel Wüthrich, Huafeng Wang, Mengyi Li, Tassanee Lerksuthirat, Sarah E. Hardison, Gordon D. Brown, Bruce Klein

**Affiliations:** ^1^ Department of PediatricsUniversity of Wisconsin School of Medicine and Public HealthUniversity of Wisconsin‐MadisonWIUSA; ^2^ Department of Internal MedicineUniversity of Wisconsin School of Medicine and Public HealthUniversity of Wisconsin‐MadisonWIUSA; ^3^ Department of Medical Microbiology and ImmunologyUniversity of Wisconsin School of Medicine and Public HealthUniversity of Wisconsin‐MadisonWIUSA; ^4^ Aberdeen Fungal GroupInstitute of Medical SciencesUniversity of AberdeenAberdeenUK

**Keywords:** Chromoblastomycosis, C‐type lectin, Fungi, Dectin‐2, Mincle, T‐cell differentiation, Th17 cell

## Abstract

Chromoblastomycosis is a chronic skin infection caused by the pigmented saprophytic mould *Fonsecaea pedrosoi*. Chronicity of infection can be broken by a coordinated innate recognition of the spores by pattern recognition receptors. While Mincle signaling via the Syk/Card9 pathway is required for fungal recognition by host cells, it is not sufficient for host control. Exogenously applied TLR agonists are necessary to promote the induction of proinflammatory cytokines and clearance of infection in vivo. Here, we investigated whether costimulation by TLR agonists fosters the development of adaptive immune responses, by examining the development of fungus‐specific T cells. Subcutaneous infection of mice with *F. pedrosoi* spores induced the activation, expansion, and differentiation of Ag‐specific CD4^+^ T cells but TLR costimulation did not further augment these T‐cell responses. The Dectin‐2/FcRγ/Card9 signaling pathway promoted the differentiation of fungus‐specific CD4^+^ T cells into Th17 cells, whereas Mincle inhibited the development of this T‐helper subset in infected mice. These results indicate differential roles for Dectin‐2 and Mincle in the generation of adaptive immune responses to *F. pedrosoi* infection.

## Introduction

Chromoblastomycosis is a chronic progressive fungal infection of mammalian skin and subcutaneous tissue that is caused by a transcutaneous inoculation of melanized fungi such as *Fonsecaea*, *Cladophialophora*, and *Phialophora* species 1, 2. *Fonsecaea pedrosoi* is the pigmented and saprophytic mold that mainly causes chromoblastomycosis. Although the disease has been diagnosed worldwide, it occurs most frequently in humid tropical and subtropical regions of America, Asia, and Africa 2, 3. It can take years between inoculation and disease development 4. Initial lesions of chromoblastomycosis manifest as erythematous papules, which gradually enlarge and become verrucous nodules and psoriasis like plaques that may extend as satellites along the lymphatics or disseminate through scratching 5. Chromoblastomycosis treatment is difficult and most therapeutic attempts provide only a modest rate of success 6, 7.

Little is known about the protective host defense mechanisms against chromoblastomycosis. Innate immunity mediated by neutrophils and macrophages is thought to be principally responsible for host protection 2, 3. However, the chronic nature of infection with *F. pedrosoi* may be due to an inappropriate innate immune response 8. *F. pedrosoi* is recognized by the C‐type lectin receptors (CLRs) Dectin‐1 and Mincle, yet fails to induce the production of proinflammatory TNF‐α by macrophages and DCs. Inflammatory responses to *F. pedrosoi* and clearance of infection can be reinstated by exogenous TLR costimulation 8. Imiquimod (TLR7 ligand) and LPS (TLR4 ligand) application augments TNF‐α production and accelerates healing in murine models 8 and in humans 9.

Since there is some evidence that CD4^+^ but not CD8^+^ T cells can mediate protective host immunity against *F. pedrosoi* infection 3, 10 we were interested in understanding whether costimulation of the TLR receptors augments adaptive CD4^+^ T‐cell responses in a similar fashion as we have reported for the innate cytokine responses 8. In order to monitor and enumerate fungus‐specific CD4^+^ T‐cell responses we evaluated whether recently generated 1807 TCR transgenic T cells, which have been shown to recognize a pan‐fungal epitope widely expressed among ascomycetes 11, can be triggered by *F. pedrosoi*. In this report, we show that *F. pedrosoi* infection activates, expands, and differentiates 1807 T cells into Th1 and Th17 cells. However, TLR costimulation does not augment these 1807 T‐cell responses. Interestingly, *F. pedrosoi* spore‐induced Th17‐cell differentiation is fostered by the Dectin‐2/FcRγ/Card9 signaling axis but inhibited by Mincle recognition.

## Results

### Infection with *F. pedrosoi* induces the development of antigen‐specific Th17 and Th1 cells

**Figure 1:**
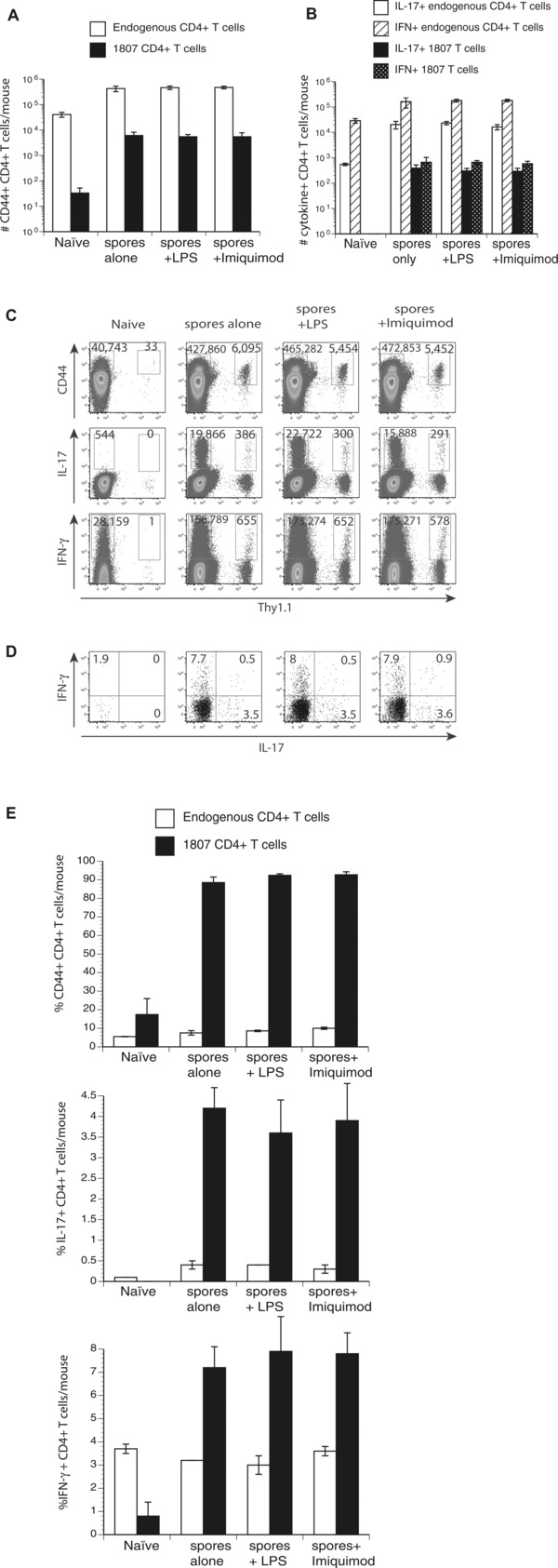
TLR costimulation does not augment T‐cell activation, expansion, and differentiation. Wild‐type C57BL6 mice received an adoptive transfer of 10^5^ CD4^+^ purified, naïve 1807 Tg cells and were infected with 2 × 10^6^ live *F. pedrosoi* spores or not. Transferred 1807 and endogenous CD4^+^ T cells were harvested from the popliteal LN and the (A) number and (E) frequencies of activated (CD44^+^), and (B) number of IL‐17‐ and IFN‐γ‐producing T cells was assessed at day 7 postinjection by flow cytometry. (C) Dot plots show concatenated samples of 4–6 mice/group. The numbers indicate the mean ± SEM of activated (CD44^hi^) and cytokine producing 1807 Tg (Thy1.1^+^) cells and endogenous CD4^+^ T cells. (D and E) The frequencies of cytokine‐producing 1807 cells were assessed by flow cytometry. Data are expressed as the mean ± SEM (*n* = 4–6 mice/group). T‐cell numbers from LPS or Imiquimod‐treated, infected mice versus nontreated infected mice were not statistically different using the Wilcoxon rank test for nonparametric data. Data are from single experiments representative of three independent experiments.

Cell‐mediated immune responses to chromoblastomycosis are poorly described. Studies in athymic nude and CD4^−/−^ mice showed poor granuloma formation, increased fungal loads and decreased DTH and IFN‐γ production supporting a role for T‐cell immune responses 10, 12. To investigate whether subcutaneous injection of *F. pedrosoi* spores activates and expands antigen‐specific CD4^+^ T cells we used TCR transgenic (Tg) 1807 cells that recognize systemic dimorphic fungi 13 and can also be activated by *F. pedrosoi* 11. We first optimized the experimental conditions to achieve increased expansion of activated 1807 cells in *F. pedrosoi* infected versus uninfected controls. Previously, we only saw about a twofold increase in the number of CD44^+^ 1807 cells for the above‐mentioned comparison 11. We reduced the number of adoptively transferred naïve 1807 cells from one million 11 to 10^5^ and used either two million or two hundred million spores for the footpad injections. Adoptive transfer of lower numbers of naive 1807 cells prior to infection increased the number of activated (CD44^+^), IL‐17 and IFN‐γ producing 1807 CD4^+^ T cells >20‐fold in mice infected with 2 million spores versus naïve mice (Fig. 1A–C). Thus, *F. pedrosoi* infection induced the generation of Ag‐specific Th17 and Th1 CD4^+^ T cells that can be tracked with adoptively transferred 1807 cells.

Increasing the inoculum 100‐fold did not yield any further activation, expansion, or differentiation (data not shown). Since not all the endogenous CD4^+^ T cells are antigen‐specific but 1807 cells are specific, we found the largest alterations in the 1807 cell population. Hence, for the studies done here we emphasize the results of transferred 1807 cells since they provided the best resolution. Endogenous CD4^+^ T cells showed a similar trend and are therefore illustrated in parallel.

### TLR costimulation does not augment Ag‐specific T‐cell development in *F. pedrosoi*‐infected mice

We have previously shown that *F. pedrosoi* spores alone failed to induce innate inflammatory responses by macrophages. The failure of innate recognition could be reinstated by TLR costimulation 8. To explore whether Ag‐specific T‐cell responses can be augmented by TLR costimulation we injected *F. pedrosoi* spore‐infected mice with LPS as described 8. Surprisingly, LPS (TLR4 agonist) and Imiquimod (TLR7 agonist) treatment did not increase the activation (CD44), expansion, and differentiation of Ag‐specific 1807 cells as indicated by the numbers and frequencies of activated (CD44^+^) and cytokine producing (IFN‐γ and IL‐17) 1807 cells (Fig. 1A–E). Thus, unlike innate inflammatory responses, the generation of adaptive cell‐mediated immune responses could not be augmented by costimulation of the TLR pathway.

### *F*. *pedrosoi* triggers Dectin‐1, Dectin‐2, Mincle, and MCL signaling and IL‐6 production

**Figure 2:**
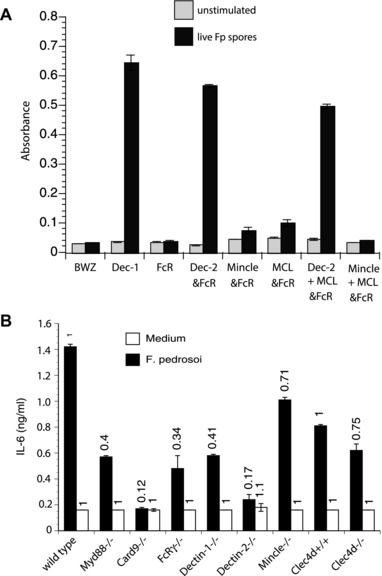
*F. pedrosoi* spores trigger Dectin‐1 and Dectin‐2 signaling and IL‐6 production. (A) BWZ cells and a subline expressing Dectin‐1‐CD3ζ (Dectin‐1), as well as B3Z cells expressing Dectin‐2, Mincle, FcRγ chain, Dectin‐2 + FcRγ, MCL + FcRγ, Dectin‐2 + MCL + FcRγ, Mincle + FcRγ or Mincle + MCL + FcRγ were stimulated with live *F. pedrosoi* spores. After 18 h, lacZ activity was measured using a colorimetric assay and expressed as OD 560/620 values. Data are expressed as the mean ± SD of duplicate wells. Data are from single experiments representative of three independent experiments. (B) Bone marrow derived dendritic cells (BMDC) were cocultured with live *F. pedrosoi* spores for 24 h and IL‐6 was detected in the supernatants by ELISA. Numbers above histogram bars indicate the *n*‐fold change versus the corresponding wild‐type control. Data are from a single experiment representative of two independent experiments.

Innate recognition of *F. pedrosoi* and *F. monophora* requires Mincle on murine macrophages and human dendritic cells 8, 14. Since *F. pedrosoi* spores triggered an adaptive T‐cell response in vivo, we sought to investigate whether surface expressed CLRs are involved in the innate recognition of the fungal spores. We cocultured live *F. pedrosoi* spores with CLR transformed B3Z T‐cell hybridoma cells expressing an NFAT‐lacZ β‐galatosidase reporter of ITAM signaling 15. In response to spore stimulation, lacZ activity was increased in reporter cells expressing Dectin‐1 and Dectin‐2 and to a lesser extent MCL (Dectin‐3, Clecsf8, or Clec4d) and Mincle, but not in cells expressing FcRγ only (Fig. 2). Since MCL has been shown to hetero‐dimerize with Dectin‐2 16 and induce coexpression of Mincle on the cell surface 17 we also measured lacZ activity in reporter cells coexpressing MCL/Dectin‐2 and MCL/Mincle. Dectin‐2/MCL and Mincle/MCL coexpressing T‐hybridoma cells produced lacZ activity comparable to Dectin‐2 and Mincle single expression cells, respectively, indicating that coexpression of MCL did not increase Dectin‐2 and Mincle induced signaling. Thus, spores recognized by Dectin‐1 and Dectin‐2 induced the strongest signaling activity, whereas Mincle and MCL recognition induced only a weak response.

To investigate whether CLR engagement by *F. pedrosoi* spores induces the production of cytokines that foster the differentiation of anti‐fungal T cells, we cocultured BMDCs and spores and measured the production of IL‐6, which is a critical priming cytokine for Th17 cells. BMDCs that lack Dectin‐1, Dectin‐2, or the downstream components of their signaling pathway (FcRγ and Card9) produced sharply reduced amounts of IL‐6 compared to wild‐type cells (Fig. 2B). The absence of MCL or Mincle on BMDCs led to a comparatively smaller reduction in IL‐6. These data inversely correlate with the reporter cell data illustrated in Fig. 2A, indicating that engagement of a CLR by *F. pedrosoi* spores leads to the production of T‐cell priming cytokines.

### Impact of the CLR/Card9 signaling pathways on *F. pedrosoi*‐induced T‐cell activation and expansion

**Figure 3:**
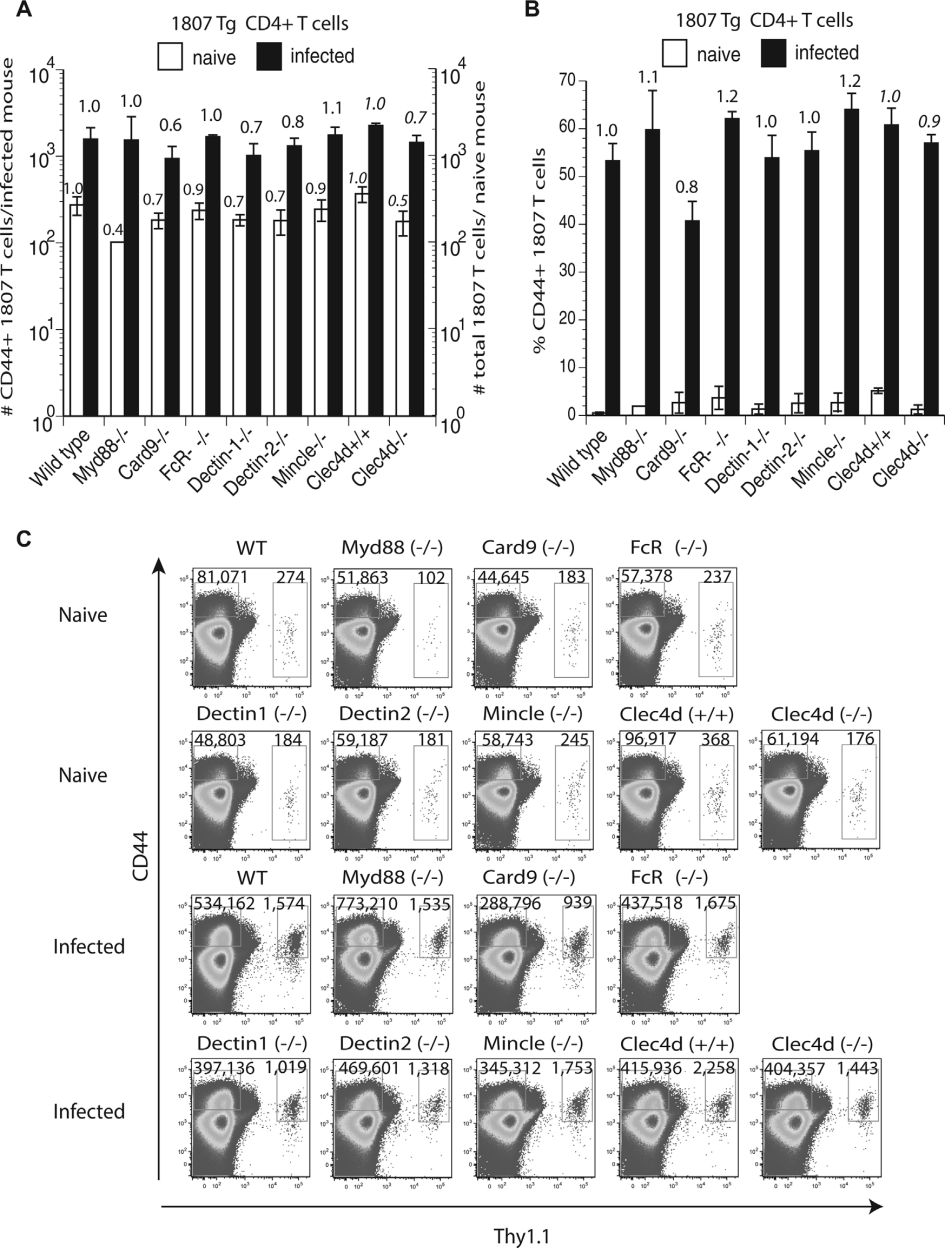
T‐cell activation and expansion is modestly regulated by Dectin‐1, Dectin‐2, MCL, and Card9. Wild type, Myd88^−/−^, Card9^−/−^, FcRγ^−/−^, Dectin‐1^−/−^, Dectin‐2^−/−^, Mincle^−/−^, and Clec4d^+/+^ and Clec4d^−/−^ mice adoptively received 10^5^ CD4^+^ purified, naïve 1807 Tg cells and were infected with 2 × 10^6^ live *F. pedrosoi* spores or not. At day 7 postinfection, the popliteal lymph nodes were harvested and the (A) number and (B) frequency of activated (CD44^+^) transferred 1807 (Thy1.1^+^) and endogenous CD4^+^ T cells was enumerated by flow cytometry. (A, B) The values over the bars in the histogram indicated the *n*‐fold change in T‐cell numbers versus the corresponding wild‐type control mice. (C) The dot plots show the sum of concatenated events from five mice per group and the values indicate the mean of endogenous and 1807 CD4^+^ T cells. Data are expressed as mean + SD of five mice per group from a single experiment representative of two independent experiments. The number and frequency of activated (CD44^+^) T cells was not statistically significant between infected knockout mice versus wild‐type controls using the Wilcoxon rank test for nonparametric data.

To investigate which innate recognition and signaling pathways are required to activate and expand antigen‐specific T cells in vivo we enumerated the number of activated (CD44^+^) 1807 T cells from the skin draining lymph nodes of *F. pedrosoi* infected wild‐type, CLR‐, and signaling adaptor‐deficient mice. Although not statistically significant, the number of activated (CD44^+^) 1807 T cells was reduced by 20–40% in infected Dectin‐1^−/−^, Dectin‐2^−/−^, Card9^−/−^, and Clec4d^−/−^ mice versus control wild‐type recipient mice (Fig. 3A+C). Little or no change in 1807 T‐cell activation and expansion was observed in infected Myd88^−/−^, FcRγ^−/−^, and Mincle^−/−^ mice versus infected control mice.

### *F*. *pedrosoi*‐induced Th17 differentiation is driven by Dectin‐2 and suppressed by Mincle recognition

**Figure 4:**
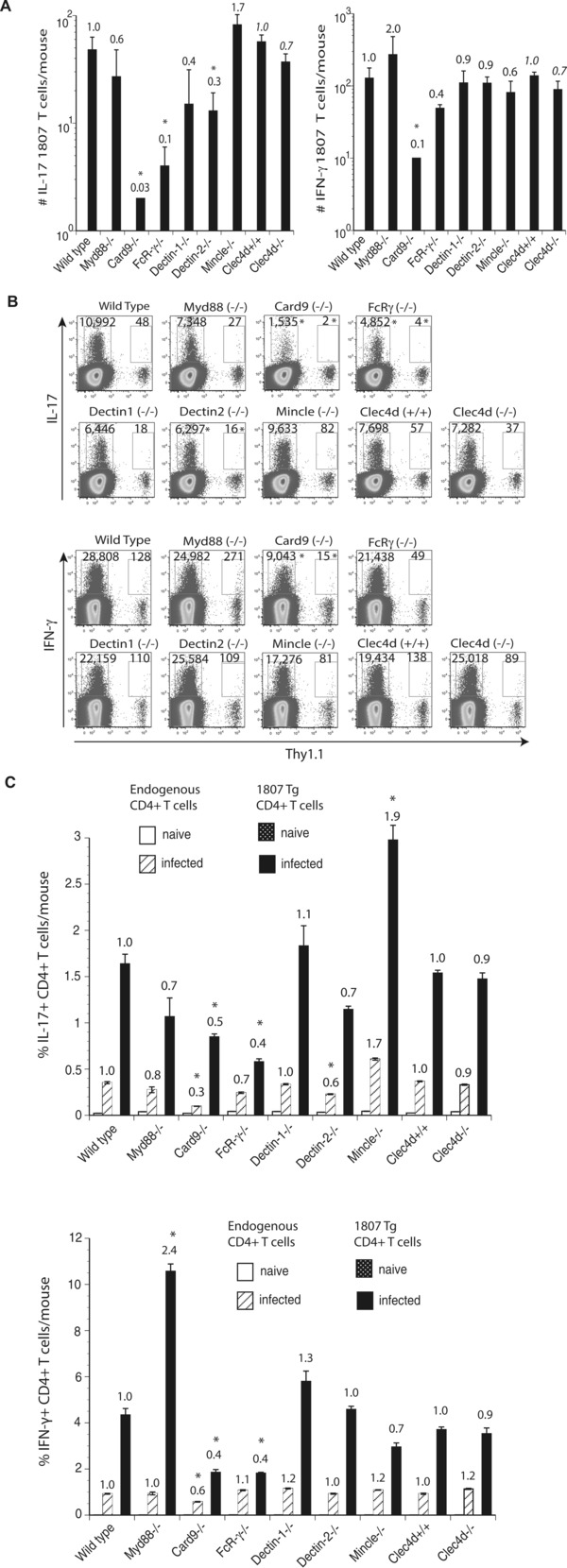
*F. pedrosoi‐*induced T‐cell differentiation requires Dectin‐2, but is suppressed by Mincle. Wild type, Myd88^−/−^, Card9^−/−^, FcRγ^−/−^, Dectin‐1^−/−^, Dectin‐2^−/−^, Mincle^−/−^, and Clec4d^+/+^ and Clec4d^−/−^ mice adoptively received 10^5^ CD4^+^ purified, naïve 1807 Tg cells and were infected with 2 × 10^6^ live *F. pedrosoi* spores or not. At day 7 postinfection, the popliteal lymph nodes were harvested and (A, B) the number and (C) frequencies of (A) IL‐17‐ and (B) IFN‐γ‐producing T cells enumerated by flow cytometry. The values over the bars in the histogram indicated the *n*‐fold change in T‐cell numbers versus the corresponding wild‐type control mice. The dot plots show the sum of concatenated events from five mice per group and the numbers indicate the mean of endogenous and 1807 CD4^+^ T cells. Data are expressed as mean + SD of five mice per group from a single experiment representative of two independent experiments. *p < 0.05 versus infected wild‐type controls using the Wilcoxon rank test for nonparametric data.

Engagement of Mincle on human DC by *F. monophora* was reported to suppress Th1 cell polarization 14. To investigate whether T‐cell differentiation was altered in response to *F. pedrosoi* infection, we enumerated the number and frequency of cytokine producing Ag‐specific T cells in the absence of the Myd88 and CLR/Card9 signaling axis. Card9^−/−^, FcRγ^−/−^, and Dectin‐2^−/−^ mice showed reduced numbers and frequencies of Th17 cells (Fig. 4A–C). Although to a lesser extent, Th1‐cell differentiation was similarly impacted in the above‐mentioned recipient strains of mice. Dectin‐1^−/−^ and Clec4d^−/−^ mice had reduced numbers but not frequencies of Th17 and Th1 cells. Interestingly, Mincle^−/−^ mice showed increased numbers and frequencies of Th17 but reduced Th1‐cell differentiation. Thus in contrast to the in vitro findings with *F. monophora*‐induced human DC and T‐cell priming 14, Mincle suppressed Th17 but not Th1‐cell differentiation in our in vivo model of murine *F. pedrosoi* infection. To summarize, the most striking observation is that the Dectin‐2/FcRγ/Card9 signaling pathway is required for Th17‐cell differentiation whereas Mincle engagement is suppressing Th17 cell polarization.

## Discussion

The underlying mechanisms of protective immunity against chronic infection with *F. pedrosoi* are poorly characterized. While elimination of fungal spores is thought to principally rely on innate immune cells such as neutrophils and macrophages there is some evidence that chronic infection with *F. pedrosoi* leads to dysregulated adaptive immunity 18. In this report, we demonstrate that (1) Dectin‐2 (and to a lesser extent Dectin‐1) mediated recognition of *F. pedrosoi* spores is largely responsible for development of Ag‐specific Th17 cells, (2) Mincle appears to inhibit the development of this T‐helper subset, and (3) TLR costimulation does not augment T‐cell responses. We chose to use a subcutaneous infection model because it closely resembles the natural route of spore infection and this model has been used to demonstrate that topical and systemic application of TLR agonists augmented innate cytokine production and reduced fungal burden 8.

In order to monitor Ag‐specific CD4^+^ T cells after *F. pedrosoi* infection, we optimized the recently generated adoptive transfer system using TCR transgenic T cells from 1807 mice to enumerate *F. pedrosoi*‐specific CD4^+^ T cells in vivo 11. Indeed, subcutaneously administered *F. pedrosoi* spores triggered about a tenfold expansion of naïve 1807 cells in vivo and are therefore a well‐suited tool to enumerate fungus‐specific T cells after spore infection.

Unlike innate TNF‐α responses 8, costimulation by multiple TLR agonists including LPS (TLR4) and Imiquimod (TLR7) did not augment spore induced T‐cell expansion, activation, and Th1 and Th17‐cell differentiation. This discrepancy could be due to a differential requirement of pattern recognition receptors (PRR) for the induction of innate cytokine production versus adaptive T‐cell responses. TNF‐α production by BMDC and macrophages following PRR costimulation was dependent on the recognition of spores by Mincle but not Dectin‐1 and Dectin‐2 as determined by using PRR‐deficient phagocytes and monoclonal blocking antibodies against PRR 8. To identify which cell surface expressed CLR recognize spores we used B3Z T‐cell hybridoma cells that express the CLRs Dectin‐1, Dectin‐2, MCL, and Mincle and the corresponding signaling adaptor FcRγ [15]. Dectin‐1 and Dectin‐2 expressing reporter cells produced the highest amounts of LacZ activity when exposed to *F. pedrosoi* spores whereas Mincle and MCL triggered low reporter activity. Thus, despite the dispensable role of Dectin‐1 and Dectin‐2 for costimulatory TNF‐α production 8, these two CLRs are highly engaged upon spore interaction. Mincle expressing B3Z reporter cells produced little LacZ activity after spore exposure. Thus, despite an indispensable role of Mincle for TNF‐α production and TLR costimulation by human DC and macrophages 8 we found little evidence for Mincle signaling using spore exposed B3Z reporter cells. This discrepancy could be due to differential levels of Mincle expression on B3Z reporter cells versus myeloid phagocytes. In murine BMDCs MCL expression is constitutive and upon engagement it can induce expression of Mincle 17. Thus, increased Mincle expression in myeloid phagocytic cells as previously described 8 could be a major factor for its more prominent and indispensable role in spore induced TNF‐α production than in the reporter assay. MCL expressed alone induced little LacZ activity whereas coexpressed with Dectin‐2 MCL did not augment Dectin‐2 induced reporter activity indicating that spores do not exhibit surface exposed ligands for MCL.

Since protective immunity to many fungi requires the generation of Th17 cells 19, 20, we were particularly interested in the identification of PRR and signaling pathways that drive the development of Th17 cells. The importance of IL‐17 responses in antifungal immunity is best documented in diverse experimental models of systemic and oral candidiasis 19, 20, 21. Th17 cells also offer protection against primary infection with *C. neoformans*, *P. carinii*, and *H. capsulatum* 22, 23, 24 and vaccine induced immunity against the systemic dimorphic fungi *B. dermatitidis*, *C. posadasii*, and *H. capsulatum* 25. To investigate which CLRs are important for the induction of a *Fonsecaea*‐specific T‐cell response we infected mice lacking Myd88, Card9, FcRγ, Dectin‐1, Dectin‐2, MCL, or Mincle and measured 1807 T‐cell expansion, activation, and differentiation. The number and frequency of IL‐17 producing 1807 and endogenous CD4^+^ T cells was reduced in Dectin‐2^−/−^, FcRγ^−/−^, and Card9^−/−^ mice. These results indicate that the Dectin‐2/FcRγ/Card9 signaling axis promotes the differentiation of *F. pedrosoi*‐specific Th17 cells. While Dectin‐1 and Mincle have previously been implicated in the innate recognition of *F. pedrosoi* spores 8, 14, in this manuscript we detected a new role for Dectin‐2 in this process. In vitro, spores were recognized by Dectin‐2 expressing reporter cells and induced signal transduction, and the lack of Dectin‐2 on BMDCs blunted IL‐6 production. In vivo, the lack of Dectin‐2 likewise reduced Th17‐cell differentiation of Ag‐specific T cells. Thus, the phenotype of Dectin‐2 in promoting Th17 cell immunity showed good fidelity with the results of the NFAT reporter cells and BMDC cocultured with spores. Only few reports describe the role of Dectin‐2 in driving Th17 cells. In mice, Dectin‐2 is required for the differentiation of Th17 cells induced by primary infection with *C. albicans* 26 and vaccine‐induced immunity to *B. dermatitidis*, *C. posadasii*, and *H. capsulatum* 15. In human dendritic cells, Dectin‐2 activation by *Candida* triggers the production of IL‐1β and IL‐23p19, which favors a Th17 response 27.

In contrast to recipient mice that lack elements of the Dectin‐2/FcRγ/Card9 signaling pathway, Mincle^−/−^ mice had increased numbers and frequencies of IL‐17^+^ 1807 cells compared to wild‐type controls, indicating that Mincle suppresses the development of Th17 cells. Since we have not enumerated fungal CFU in Mincle^−/−^ and other CLR^−/−^ mice, we cannot rule out the possibility that a difference in fungal burden could have impacted the development of adaptive immunity. However, independent of the fungal burden only T‐cell differentiation and not activation and expansion was affected in Mincle^−/−^ versus wild‐type mice. The inhibition of in vivo Th17 responses by Mincle were not reflected by the lack of reporter activity by the Mincle expressing T‐cell hybridoma cells and increased IL‐6 production by Mincle^−/−^ BMDCs in vitro. It is possible that Mincle is part of a receptor complex that regulates Th17 responses, thus the reporter assay may not reflect this complexity. Although IL‐6 is a crucial Th17‐cell priming cytokine, it may not reflect all aspects of Th17‐cell differentiation in our model and additional factors from other cells participating in antigen processing and presentation may be needed to drive this T helper subset. The role of Mincle in mediating anti‐microbial and anti‐fungal immunity has been recently investigated. In several murine models Mincle was found to be an activating receptor upon ligand engagement and its expression can be induced via triggering of MCL (Dectin‐3) 17. Mincle is an FcRγ‐coupled activating receptor that recognizes cord factor, also called trehalose‐6,6’‐dimycolate (TDM) that is a mycobacterial glycolipid and is used as a vaccine adjuvant 28, 29. Thus Mincle is a key regulator for TDM and its synthetic derivate TDB induced therapeutic Th1 and Th17 adjuvanticity 30. Mincle is also an activating receptor for the pathogenic fungus *Malassezia*, recognizing α‐mannose 31, glyceroglycolipid, and unique mannosyl fatty acids linked to mannitol as ligands 32. Following *F. pedrosoi* infection in mice, Mincle is required but not sufficient for innate recognition of the spores 8. In contrast, in human DC TLR4‐induced IL‐12p70 production and subsequent Th1‐cell differentiation was suppressed by Mincle 14. Deficiency of Th1 effector responses due to Mincle‐mediated suppression of IL‐12p35 expression was found in response to infection with *F. monophora*, *F. pedrosoi*, *C. carrionii*, and *F. compacta* suggesting a common mode of immune evasion in humans. Our in vivo findings in mice would argue that Mincle‐mediated suppression is not restricted to humans only and the development of Th1 cells. Instead of being an activator of Th17 cells as in the case of TDM and TDB induced adjuvanticity 30, we found Mincle to be a suppressor of *F. pedrosoi* induced Th17 cells. Thus the overall adaptive immune response to *F. pedrosoi* spores in the subcutaneous murine infection model is determined by the cooperation of activating Dectin‐2 and regulating Mincle signaling.

## Materials and methods

### Ethics statement

All animal procedures were performed in accordance with the recommendations in the Guide for the Care and Use of Laboratory Animals of the National Institutes of Health. Care was taken to minimize animal suffering. The work was done with the approval of the IACUC of the University of Wisconsin‐Madison.

### Mouse strains

Inbred wild‐type C57BL/6 and congenic B6. PL‐Thy1^a^/Cy (stock #00406) mice carrying the Thy 1.1 allele were obtained from Jackson Laboratories, Bar Harbor, ME. B6.129P2‐*Fcer1g^tm1Rav^* N12 mice (model # 583) that lack FcRγ were purchased from Taconic. *Blastomyces*‐specific TCR Tg 1807 mice were generated in our lab and were backcrossed to congenic Thy1.1^+^ mice as described elsewhere 13. Card9^−/−^ 33, Dectin‐1^−/‐^ 34, Dectin‐2^−/‐^ 26, Clec4d^+/+^, and Clec4d^−/−^(obtained from the Consortium of Functional Genomics), Mincle^−/−^ 35 and Myd88^−/−^ 36 mice were bred at our facility. All mice were 7–8 weeks at the time of experiments. Mice were housed and cared for according to guidelines of the University of Wisconsin Animal Care Committee, who approved all aspects of this work.

### *F. pedrosoi* growth conditions and in vivo model

*F. pedrosoi* ATCC 46428 was maintained on potato agar plates and liquid potato broth containing 50 μg/mL chloramphenicol in a shaking incubator at 30ºC for in vitro and in vivo assays. The conidia were filtered to remove hyphae and washed with PBS before use. To measure *F. pedrosoi*‐specific CD4^+^ T‐cell responses in vivo, mice were injected i.v. with a single cell suspension of 10^5^ magnetic bead purified CD4^+^ cells from 1807 Tg Thy1.1^+^ mice 13. Subsequently, mice were subcutaneously injected with 2 × 10^6^ or 2 × 10^8^ conidia of *F. pedrosoi* into one footpad. For TLR costimulation, 100 ng LPS was administered i.p. at day 3 postinfection. This concentration and route of delivery induced a 20‐fold increase in TNF‐α transcript in PBMC versus naïve mice indicating it was functional. All mice were sacrificed at day 7 and the popliteal lymph nodes harvested for T‐cell analysis. An aliquot of lymph node cells was stained with mAbs directed against the following surface markers: CD4, CD8, Thy1.1, CD44, and B220 (as a dump marker). For intracellular cytokine staining T cells were stimulated with anti‐CD3 and anti‐CD28 mAb in the presence of Golgi‐Stop (BD Pharmingen) for 4–6 h. Stimulated T cells were stained for surface markers, fixed, and permeabilized using the Cytofix/Cytoperm kit (BD Pharmingen) and stained with anti‐IFN‐γ and anti‐IL‐17 antibodies. All mAbs were obtained from BD PharMingen (San Diego, CA) and eBioscience (San Diego, CA) and cytometry data were gathered with a LSRII flow cytometer (BD Biosciences, San Jose). Data were analyzed by FlowJo software (Tree Star, Ashland, OR). The number of 1807 and endogenous CD4^+^ T cells was calculated by multiplying the percentage of Thy1.1^+^ and Thy1.1^−^ CD4^+^ cells by the number of viable cells as determined by trypan blue dye exclusion.

### CLR reporter cells and stimulation by *F. pedrosoi* spores

B3Z cells carrying an NFAT‐lacZ reporter construct have been described preciously 37 and were provided by Dr. Nilabh Shastri (University of California, Berkeley, CA). To generate MCL expressing reporter cells, MCL was cloned from mouse BMDC cDNA with a C‐terminal Flag tag into the EcoRI/NotI sites of a retroviral vector pMX‐IRES‐hCD8 (kindly provided by Dr. Sho Yamasaki). B3Z cells were retrovirally transduced with pMX‐mMCL‐Flag‐IRES‐hCD8, purified using anti‐hCD8 microbeads (Miltenyi Biotec), and verified by cell surface staining and FACS analysis using an anti‐mouse MCL mAb (a gift from Dr. Sho Yamasaki) and an anti‐Flag mAb (Sigma). The generation of Mincle expressing reporter cells was described elsewhere 15. B3Z cells expressing Dectin‐2 and/or a wild type or a signaling‐defective FcRγ chain that carries two tyrosine‐to‐phenylalanine mutations within the ITAM motif 38, as well as BWZ cells expressing Dectin‐1‐CD3ζ (the extracellular domain of mouse Dectin‐1 fused to CD3ζ intracellular tail) 39 and the parent BWZ cells 40, were a generous gift from Dr. Caetano Reis e Sousa (London Research Institute, United Kingdom) and have previously been used in our lab 15.

For B3Z/BWZ cell stimulation, 10^5^ B3Z/BWZ cells/well in a 96‐well plate were incubated for 18 h with 3 × 10^6^ live *F. pedrosoi* spores. LacZ activity was measured in total cell lysates using CPRG (Roche) as a substrate. OD 560 was measured using OD 620 as a reference.

### Statistical analysis

Differences in the number and percentage of activated, proliferating, or cytokine‐producing T cells were analyzed using the Wilcoxon rank test for nonparametric data or the *t*‐test (using GraphPad Prism) when data were normally distributed 41. A *p* value < 0.05 is considered statistically significant.

## Conflict of interest

The authors declare no financial or commercial conflict of interest.

**Abbreviations**
CLR: C‐type lectin receptor
PRR: pattern recognition receptor
TDM: trehalose‐6,6’‐dimycolate

## Supporting information

As a service to our authors and readers, this journal provides supporting information supplied by the authors. Such materials are peer reviewed and may be re‐organized for online delivery, but are not copy‐edited or typeset. Technical support issues arising from supporting information (other than missing files) should be addressed to the authors.

Peer review correspondence

## References

[1] Queiroz‐Telles, F. , Esterre, P. , Perez‐Blanco, M. , Vitale, R. G. , Salgado, C. G. and Bonifaz, A. , Chromoblastomycosis: an overview of clinical manifestations, diagnosis and treatment. Med. Mycol. 2009 47: 3–15. 19085206 10.1080/13693780802538001

[2] Ameen, M. , Chromoblastomycosis: clinical presentation and management. Clin. Exp. Dermatol. 2009 34: 849–854. 19575735 10.1111/j.1365-2230.2009.03415.x

[3] Santos, A. L. , Palmeira, V. F. , Rozental, S. , Kneipp, L. F. , Nimrichter, L. , Alviano, D. S. , Rodrigues, M. L. and Alviano, C. S. , Biology and pathogenesis of *Fonsecaea pedrosoi*, the major etiologic agent of chromoblastomycosis. FEMS Microbiol. Rev. 2007 31: 570–591. 17645522 10.1111/j.1574-6976.2007.00077.x

[4] Brandt, M. E. and Warnock, D. W. , Epidemiology, clinical manifestations, and therapy of infections caused by dematiaceous fungi. J. Chemother. 2003 15 (Suppl 2): 36–47. 14708965 10.1179/joc.2003.15.Supplement-2.36

[5] Ricard‐Blum, S. , Hartmann, D. J. and Esterre, P. , Monitoring of extracellular matrix metabolism and cross‐linking in tissue, serum and urine of patients with chromoblastomycosis, a chronic skin fibrosis. Eur. J. Clin. Invest. 1998 28: 748–754. 9767374 10.1046/j.1365-2362.1998.00335.x

[6] Bonifaz, A. , Martinez‐Soto, E. , Carrasco‐Gerard, E. and Peniche, J. , Treatment of chromoblastomycosis with itraconazole, cryosurgery, and a combination of both. Int. J. Dermatol. 1997 36: 542–547. 9268758 10.1046/j.1365-4362.1997.00085.x

[7] Esterre, P. and Queiroz‐Telles, F. , Management of chromoblastomycosis: novel perspectives. Curr. Opin. Infect. Dis. 2006 19: 148–152. 16514339 10.1097/01.qco.0000216625.28692.67

[8] da Gloria Sousa, M. , Reid, D. M. , Schweighoffer, E. , Tybulewicz, V. , Ruland, J. , Langhorne, J. , Yamasaki, S. et al., Restoration of pattern recognition receptor costimulation to treat chromoblastomycosis, a chronic fungal infection of the skin. Cell Host Microbe 2011 9: 436–443. 21575914 10.1016/j.chom.2011.04.005 PMC3098964

[9] de Sousa Mda, G. , Belda, W., Jr. , Spina, R. , Lota, P. R. , Valente, N. S. , Brown, G. D. , Criado, P. R. et al., Topical application of imiquimod as a treatment for chromoblastomycosis. Clin. Infect Dis. 2014 58: 1734–1737. 24633683 10.1093/cid/ciu168 PMC4036686

[10] Teixeira de Sousa Mda, G. , Ghosn, E. E. and Almeida, S. R. , Absence of CD4+ T cells impairs host defence of mice infected with *Fonsecaea pedrosoi* . Scand. J. Immunol. 2006 64: 595–600. 17083615 10.1111/j.1365-3083.2006.01846.x

[11] Wüthrich, M. , Brandhorst, T. T. , Sullivan, T. , Filutowicz, H. , Stewart, D. , Shen, Z. , Ostroff, G. R. et al., Calnexin induces expansion of antigen‐specific CD4 T cells that confer immunity to fungal ascomycetes via conserved epitopes. Cell Host Microbe 2015 17: 452–465. 25800545 10.1016/j.chom.2015.02.009 PMC4484745

[12] Ahrens, J. , Graybill, J. R. , Abishawl, A. , Tio, F. O. and Rinaldi, M. G. , Experimental murine chromomycosis mimicking chronic progressive human disease. Am. J. Trop. Med. Hyg. 1989 40: 651–658. 2742041 10.4269/ajtmh.1989.40.651

[13] Wüthrich, M. , Ersland, K. , Sullivan, T. , Galles, K. and Klein, B. S. , Fungi subvert vaccine T cell priming at the respiratory mucosa by preventing chemokine‐induced influx of inflammatory monocytes. Immunity 2012 36: 680–692. 22483803 10.1016/j.immuni.2012.02.015 PMC3334432

[14] Wevers, B. A. , Kaptein, T. M. , Zijlstra‐Willems, E. M. , Theelen, B. , Boekhout, T. , Geijtenbeek, T. B. and Gringhuis, S. I. , Fungal engagement of the C‐type lectin mincle suppresses dectin‐1‐induced antifungal immunity. Cell Host Microbe 2014 15: 494–505. 24721577 10.1016/j.chom.2014.03.008

[15] Wang, H. , Lebert, V. , Hung, C. Y. , Galles, K. , Saijo, S. , Lin, X. , Cole, G. T. et al., C‐type lectin receptors differentially induce th17 cells and vaccine immunity to the endemic mycosis of North America. J. Immunol. 2014 192: 1107–1119. 24391211 10.4049/jimmunol.1302314 PMC3910401

[16] Zhu, L. L. , Zhao, X. Q. , Jiang, C. , You, Y. , Chen, X. P. , Jiang, Y. Y. , Jia, X. M. et. al., C‐type lectin receptors dectin‐3 and dectin‐2 form a heterodimeric pattern‐recognition receptor for host defense against fungal infection. Immunity 2013 39: 324–334. 23911656 10.1016/j.immuni.2013.05.017

[17] Miyake, Y. , Toyonaga, K. , Mori, D. , Kakuta, S. , Hoshino, Y. , Oyamada, A. , Yamada, H. et al., C‐type lectin MCL is an FcRgamma‐coupled receptor that mediates the adjuvanticity of mycobacterial cord factor. Immunity 2013 38: 1050–1062. 23602766 10.1016/j.immuni.2013.03.010

[18] Mazo Favero Gimenes, V. , Da Gloria de Souza, M. , Ferreira, K. S. , Marques, S. G. , Goncalves, A. G. , Vagner de Castro Lima Santos, D. , Pedroso e Silva Cde, M. et al., Cytokines and lymphocyte proliferation in patients with different clinical forms of chromoblastomycosis. Microbes Infect 2005 7: 708–713. 15848277 10.1016/j.micinf.2005.01.006

[19] Leibundgut‐Landmann, S. , Wüthrich, M. and Hohl, T. M. , Immunity to fungi. Curr. Opin. Immunol. 2012 24: 449–458. 22613091 10.1016/j.coi.2012.04.007 PMC3538869

[20] Wüthrich, M. , Deepe, G. S., Jr. and Klein, B. , Adaptive immunity to fungi. Annu. Rev. Immunol. 2012 30: 115–148. 22224780 10.1146/annurev-immunol-020711-074958 PMC3584681

[21] Conti, H. R. , Shen, F. , Nayyar, N. , Stocum, E. , Sun, J. N. , Lindemann, M. J. , Ho, A. W. et al., Th17 cells and IL‐17 receptor signaling are essential for mucosal host defense against oral candidiasis. J. Exp. Med. 2009 206: 299–311. 19204111 10.1084/jem.20081463 PMC2646568

[22] Rudner, X. L. , Happel, K. I. , Young, E. A. and Shellito, J. E. , Interleukin‐23 (IL‐23)‐IL‐17 cytokine axis in murine *Pneumocystis carinii* infection. Infect Immun. 2007 75: 3055–3061. 17403873 10.1128/IAI.01329-06 PMC1932856

[23] Wozniak, K. L. , Hardison, S. E. , Kolls, J. K. and Wormley, F. L. , Role of IL‐17A on resolution of pulmonary *C. neoformans* infection. PLoS One 2011 6: e17204. 21359196 10.1371/journal.pone.0017204 PMC3040760

[24] Deepe, G. S., Jr. and Gibbons, R. S. , Interleukins 17 and 23 influence the host response to *Histoplasma capsulatum* . J. Infect Dis. 2009 200: 142–151. 19469707 10.1086/599333 PMC2715335

[25] Wüthrich, M. , Gern, B. , Hung, C. Y. , Ersland, K. , Rocco, N. , Pick‐Jacobs, J. , Galles, K. et al., Vaccine‐induced protection against 3 systemic mycoses endemic to North America requires Th17 cells in mice. J. Clin. Invest. 2011 121: 554–568. 21206087 10.1172/JCI43984 PMC3026727

[26] Saijo, S. , Ikeda, S. , Yamabe, K. , Kakuta, S. , Ishigame, H. , Akitsu, A. , Fujikado, N. et al., Dectin‐2 recognition of alpha‐mannans and induction of Th17 cell differentiation is essential for host defense against *Candida albicans* . Immunity 2010 32: 681–691. 20493731 10.1016/j.immuni.2010.05.001

[27] Gringhuis, S. I. , Wevers, B. A. , Kaptein, T. M. , van Capel, T. M. , Theelen, B. , Boekhout, T. , de Jong, E. C. et al., Selective C‐Rel activation via Malt1 controls anti‐fungal T(H)‐17 immunity by dectin‐1 and dectin‐2. PLoS Pathog. 2011 7: e1001259. 21283787 10.1371/journal.ppat.1001259 PMC3024268

[28] Ishikawa, E. , Ishikawa, T. , Morita, Y. S. , Toyonaga, K. , Yamada, H. , Takeuchi, O. , Kinoshita, T. et al., Direct recognition of the mycobacterial glycolipid, trehalose dimycolate, by C‐type lectin Mincle. J. Exp. Med. 2009 206: 2879–2888. 20008526 10.1084/jem.20091750 PMC2806462

[29] Yamasaki, S. , Ishikawa, E. , Sakuma, M. , Hara, H. , Ogata, K. and Saito, T. , Mincle is an ITAM‐coupled activating receptor that senses damaged cells. Nat. Immunol. 2008 9: 1179–1188. 18776906 10.1038/ni.1651

[30] Schoenen, H. , Bodendorfer, B. , Hitchens, K. , Manzanero, S. , Werninghaus, K. , Nimmerjahn, F. , Agger, E. M. et al., Cutting edge: Mincle is essential for recognition and adjuvanticity of the mycobacterial cord factor and its synthetic analog trehalose‐dibehenate. J. Immunol. 2010 184: 2756–2760. 20164423 10.4049/jimmunol.0904013 PMC3442336

[31] Yamasaki, S. , Matsumoto, M. , Takeuchi, O. , Matsuzawa, T. , Ishikawa, E. , Sakuma, M. , Tateno, H. et al., C‐type lectin Mincle is an activating receptor for pathogenic fungus, Malassezia. Proc. Natl. Acad. Sci. USA 2009 106: 1897–1902. 19171887 10.1073/pnas.0805177106 PMC2644135

[32] Ishikawa, T. , Itoh, F. , Yoshida, S. , Saijo, S. , Matsuzawa, T. , Gonoi, T. , Saito, T. et al., Identification of distinct ligands for the C‐type lectin receptors Mincle and dectin‐2 in the pathogenic fungus Malassezia. Cell Host Microbe 2013 13: 477–488. 23601109 10.1016/j.chom.2013.03.008

[33] Hsu, Y. M. , Zhang, Y. , You, Y. , Wang, D. , Li, H. , Duramad, O. , Qin, X. F. et al., The adaptor protein CARD9 is required for innate immune responses to intracellular pathogens. Nat. Immunol. 2007 8: 198–205. 17187069 10.1038/ni1426

[34] Taylor, P. R. , Tsoni, S. V. , Willment, J. A. , Dennehy, K. M. , Rosas, M. , Findon, H. , Haynes, K. et al., Dectin‐1 is required for beta‐glucan recognition and control of fungal infection. Nat. Immunol. 2007 8: 31–38. 17159984 10.1038/ni1408 PMC1888731

[35] Wells, C. A. , Salvage‐Jones, J. A. , Li, X. , Hitchens, K. , Butcher, S. , Murray, R. Z. , Beckhouse, A. G. et al., The macrophage‐inducible C‐type lectin, mincle, is an essential component of the innate immune response to *Candida albicans* . J. Immunol. 2008 180: 7404–7413. 18490740 10.4049/jimmunol.180.11.7404

[36] Adachi, O. , Kawai, T. , Takeda, K. , Matsumoto, M. , Tsutsui, H. , Sakagami, M. , Nakanishi, K. et al., Targeted disruption of the MyD88 gene results in loss of IL‐1‐ and IL‐18‐mediated function. Immunity 1998 9: 143–150. 9697844 10.1016/s1074-7613(00)80596-8

[37] Karttunen, J. , Sanderson, S. and Shastri, N. , Detection of rare antigen‐presenting cells by the lacZ T‐cell activation assay suggests an expression cloning strategy for T‐cell antigens. Proc. Natl. Acad. Sci. USA 1992 89: 6020–6024. 1378619 10.1073/pnas.89.13.6020 PMC402130

[38] Robinson, M. J. , Osorio, F. , Rosas, M. , Freitas, R. P. , Schweighoffer, E. , Gross, O. , Verbeek, J. S. et al., Dectin‐2 is a Syk‐coupled pattern recognition receptor crucial for Th17 responses to fungal infection. J. Exp. Med. 2009 206: 2037–2051. 19703985 10.1084/jem.20082818 PMC2737172

[39] Sancho, D. , Joffre, O. P. , Keller, A. M. , Rogers, N. C. , Martinez, D. , Hernanz‐Falcon, P. , Rosewell, I. et al., Identification of a dendritic cell receptor that couples sensing of necrosis to immunity. Nature 2009 458: 899–903. 19219027 10.1038/nature07750 PMC2671489

[40] Sanderson, S. and Shastri, N. , LacZ inducible, antigen/MHC‐specific T cell hybrids. Int. Immunol. 1994 6: 369–376. 8186188 10.1093/intimm/6.3.369

[41] Fisher, L. D. and van Belle, G. , Biostatistics: A Methodology for the Health Sciences. John Wiley & Sons, New York, 1993, pp. 611–613.

