# Supplementary material for: Fonsecaea pedrosoi‐induced Th17‐cell differentiation in mice is fostered by Dectin‐2 and suppressed by Mincle recognition
Source: Eur J Immunol. 2015 Jul 28;45(9):2542–52. doi: 10.1002/eji.201545591 (PMC4562893; doi:10.1002/eji.201545591)
Supplement: Supplementary file 1 — Peer review correspondence [file EJI-45-2542-s001.pdf]

Peer review correspondence

European Journal of Immunology

F. pedrosoi-induced Th17-cell differentiation in mice is fostered by Dectin-2 and suppressed by Mincle recognition

Marcel Wüthrich, Huafeng Wang, Mengyi Li, Tassanee Lerksuthirat, Sarah E. Hardison, Gordon D. Brown and Bruce Klein

Corresponding author: Marcel Wüthrich, Department of Pediatrics, University of Wisconsin, Microbial Sciences Building, 1550 Linden Drive, Madison, WI, United States of America

---

Review Timeline:	Submission date:	16 February 2015
	First editorial decision:	23 March 2015
	First revision received:	6 May 2016
	Second editorial decision:	3 June 2015
	Second revision received:	8 June 2015
	Accepted:	29 June 2015

Handling Executive Committee members: Prof. Maria Yazdanbakhsh

Please note that the correspondence below does not include the standard editorial instructions regarding preparation and submission of revised manuscripts, only the scientific revisions requested and addressed.

First Editorial Decision – 23 March 2015

Dear Dr. Wuethrich,

Manuscript ID eji.201545591 entitled "Engagement of C-type lectin receptors by Fonsecaea pedrosoi spores induces differential T cell immunity" which you submitted to the European Journal of Immunology has been reviewed.

The comments of the referees are included at the bottom of this letter. Even though ref.#2 has suggested rejection, the other two referees and the Executive Editor would like to see a revised version of your manuscript. The revised manuscript, which should takes into account the comments of all the referees, will be reconsidered for publication.

European Journal of Immunology

You should also pay close attention to the editorial comments included below. **In particular, please edit your figure legends to follow Journal standards as outlined in the editorial comments. Failure to do this will result in delays in the re-review process.**

Please note that submitting a revision of your manuscript does not guarantee eventual acceptance, and that your revision will be re-reviewed by the referee(s) before a decision is rendered.

If the revision of the paper is expected to take more than three months, please inform the editorial office. Revisions taking longer than six months may be assessed by new referee(s) to ensure the relevance and timeliness of the data.

Once again, thank you for submitting your manuscript to European Journal of Immunology and we look forward to receiving your revision.

Yours sincerely,
Laura Soto Vazquez

On behalf of Prof. Maria Yazdanbakhsh

Editorial Office
European Journal of Immunology
e-mail: ejied@wiley.com
www.eji-journal.eu

****

Reviewer: 1

Comments to the Author

In this manuscript, Wuthrich et al. make use of a novel TCR-transgenic mouse generated in their lab, which is specific for an antigenic epitope that is conserved in several different ascomycota, to assess the regulation of the T cell response against Fonsecaea pedrosoi, a fungus that can case chromoblastomycosis in humans. While the authors described previously that innate recognition of F. pedrosoi is mediated via Mincle/Syk/Card9, they report here that Mincle is redundant for Th17 induction or even inhibits it, while Dectin-2 promotes the response. From this, they conclude that Mincle and Dectin-2 play differential roles in the innate and adaptive response to F. pedrosoi. Furthermore, they explore the possibility that TLR signaling modulates T cell activation and differentiation, as they showed previously

European Journal of Immunology

that TLR signaling promotes innate protection against this fungus. Although this manuscript reveals interesting aspects about the complex orchestration of the host response against F. pedrosoi, it leaves open some important questions.

Specific comments:

1. Using a subcutaneous infection model of F. pedrosoi, the authors observed increased activation (as determined by CD44+ expression) and effector differentiation (IFN-g and IL-17 production) of endogenous and 1807 TCR-transgenic CD4+ T cells in response to infection. Adoptively transferred 1807 T cells appear uniformly CD44+ already prior to infection (Fig. 1C), raising the question whether the increase in cell number upon infection reflects bona fide T cell activation or rather expansion of pre-activated/memory T cells, given that adoptively transferred cells were not restricted to naïve (CD44-, CD62L+) cells. Monitoring T cell proliferation by CFSE-dilution may thus provide a more sensitive readout for the T cell responsiveness in the different conditions. Also, in Fig. 3B, the CD44+ population within the Thy1.1 TCR-tg cells is gated differently for the naïve and the infected samples, which greatly biases the results.

2. Cytokine production was assessed after polyclonal re-stimulation, which leaves open the question to what extent the endogenous response is actually specific for the fungus.

3. In Fig. 1, co-stimulation of F. pedrosoi-infected mice with LPS did not lead to enhanced T cells responses. If anything, it led to a reduction of the antifungal response, something the authors did not comment on. The question arises whether the timing of administering the LPS was ideal. Also, the authors should provide a control for the activity of the LPS (e.g. measuring TNF in the serum).

4. The authors show that Th17 induction in response to F. pedrosoi is not reduced in Mincle-/- mice (Fig. 3 and 4) and that F. pedrosoi does not bind to Mincle, but to Dectin-2 (Fig. 2). These data are conflicting with previous findings from the authors, which showed that absence of Mincle but not Dectin-2 signaling led to a reduced inflammatory response to F. pedrosoi (de Gloria Sousa, Cell Host and Microbe, 2011). To what extent are the differences between the two reports explained by differences in the experimental systems used? While in this study they used a BWZ assay to assess binding of F. Pedrosoi to Mincle (and Dectin-2), they analyzed DCs in their previous study for their capacity to produce cytokines in absence of Mincle (or Dectin-2) signaling. Because T cell differentiation depends on APC-derived cytokines, cytokine production by APCs in response to F. pedrosoi is an important parameter for explaining the observed effects in Figure 3 and 4. Therefore, to support their current data (and possibly to clarify discrepancies arising from the two studies), the authors should look at relevant APCs in the infected organ and/or in the draining LN of infected mice and compare their cytokine profile in WT, Mincle-/- and Dectin-2-/- mice.

5. Linked to the above, the authors should check for fungal load in Mincle-/- mice in their model. If Mincle-/- mice have a defective innate control and thus increased burden of F. pedrosoi (as described by de Gloria Sousa et al, Cell Host and Microbe, 2011), this may affect the extent of the adaptive response. Increased fungal load (and thus increased availability of antigen) may lead to an enhanced adaptive response, and the contribution of Mincle to T cell priming may thereby be masked. Similarly, the effect of Card9 and FcRg on T cell priming in response to F. pedrosoi may be underestimated. Similarly, I wonder

to what extent the increased Th1 response in MyD88-/- mice impacts on the reduced Th17 response in these mice, while the increased Th17 response in Mincle-/- may affect the Th1 subset (or vice versa).
6. Th17 induction is incomplete in both, Card9-/- and MyD88-/-, which suggest that additional innate pathways may contribute, or that the Dectin-2/Card9 and the MyD88 pathways are partially redundant. This could be tested experimentally e.g. by generating double-deficient mice or by blocking Dectin-2 in MyD88-/- mice.
7. How do the authors conciliate their finding that 1807 T cells were reduced in Decint-2-/-, but not FcRg-/- mice (Fig. 3) with the essential role of FcRg for Dectin-2 surface expression?

Minor points:
The sentence 'Dectin-2 mediated recognition of F. pedrosoi spores is REQUIRED for development of Ag-specific Th17 cells' (page 9, 1st paragraph) is overstated and should be changed. There is only a partial reduction of Th17 induction observed in Dectin-2-deficient mice (Fig. 4).
On page 10, towards the end of the 1st paragraph, the authors say 'Thus, increased Mincle expression… in the reporter assay in which Mincle and MCL were NOT co-expressed.', but they actually included a condition of Mincle and MCL coexpression in Fig. 2.
On page 10, 2nd paragraph, reference 21 is not appropriate for 'IL-17 responses in antifungal immunity in models of systemic candidiasis (ref. 21 does not show any data on systemic candidiasis).

Reviewer: 2

Comments to the Author
This study describes investigates the role of C-type lectin receptors (CLR) in adaptive immune responses to F. pedrosoi. Previously, the authors have shown that TLR co-stimulation is required to induce innate immunity to F. pedrosoi, since Mincle activation alone is not sufficient for host defence. Here the authors show that TLR-costimulation does not affect adaptive immunity as assessed by Ag-specific Th17 activation. Using CLR deficient mice, they show that the immune responses (Th17) are severely impaired in dectin-1 and dectin-2 deficient mice, whereas Mincle deficient mice have higher Th17 responses. The authors conclude that dectin-1 and -2 are required for adaptive immunity to F. pedrosoi whereas Mincle seems to suppress Th17 responses.

The lack of TLR co-stimulation is striking since the authors have previously shown that co-stimulation is required for induction of innate responses. These data suggest that F. pedrosoi can induce cytokines via dectin-1 and -2, in contrast to the previous study. In vitro stimulation of DC from different CLR deficient mice would support the suggested roles for the CLR in infections especially the role for MCL and Mincle.

Major concerns

Figure 1. The use of fungus-specific T cells is interesting but it is not clear whether the induction of the 1807 T cells is fungus specific. Controls should be included to demonstrate that the increased proliferation is not due to general inflammation. The induction of endogenous T cells suggests non-specific activation.

Figure 2. F. pedrosoi has been shown to bind Mincle but using the cell-line, Mincle and MCL activation are rather small, even though the authors suggest that Mincle is important in suppressing Th17 responses. The small increase in activation does not explain the strong effect on Th17 responses. this should be further investigated. In vitro stimulations using Mincle deficient mice would provide a more clear answer. This is important since previous studies using mycobacterial ligand strongly suggest that Mincle enhances/induces Th17 responses.

Figure 3. the T cells in naïve mice are already different in the CLR deficient mice. This could impact the observed proliferation during infection. How did the authors control for this? Mincle and Clec4d deficient mice have already higher 1807 T cell-numbers in naïve mice. Could this affect the observed Th17 responses observed in Figure 4. In vitro stimulations are required to support the hypothesis of the authors concerning dectin-1, -2 and mincle in infection.

Reviewer: 3

Comments to the Author

This manuscript primarily addressed the question if co-stimulation by TLR agonists fosters the development innate and also adaptive immune responses to F pedrosoi, by promoting development of T-cells specific to the fungus. While Fp Ag specific CD4+ T-cells are induced upon infection, from the initial set of experiments it seems that TLR agonists do not promote the T-cell development further. The authors proceeded to carry out basic experiments to investigate which C-type lectins, some of which had been implicated in the innate responses to F pedrosoi, may be involved in augmenting T-cell responses. It is concluded that among a set of lectins possible involved in recognition of Fp antigens, Dectin-2 via the FcRg/Card9 pathway promotes differentiations of CD4+ into Th17 cells whereas Mincle engagement inhibits.

In general this manuscript contains interesting information, as far as I am aware as a non-specialist in fungal infection immunology, but the way the data are presented now and the questions the authors attempt to answer are not always clear to me. To the primary question - do TLR agonists stimulate/augment fungus specific T cell development - the answer quite clearly no. But the rest of paper becomes less clear as it twists in a slightly different direction (with some questions about the data below), with a more preliminary and open end.

European Journal of Immunology

Specific questions/comments:

Fig 1. The statistical relevance indication as mentioned in the legend is lacking in the figure. Why are frequencies not given, as in Fig 4C, this would be helpful to see what is going on more easy.

In relation to figure 2, except for Mincle which was implied in Fp immunity previously, what is the rationale of investigating this specific set of C-type lectin signalling

From Fig 2. is was concluded that Dectin-1 and -2 induce strong signalling and Mincle only weak. Mincle is very weak in fact, surprising in view of the later conclusions about Mincle involvement in Fp immunity. Or is it possible that Dectin is more highly expressed on the cells, hence gives a higher signal? A positive control for each lectin would have been necessary to draw conclusions about relative strong or weak signalling.

Fig 3A. The bar in the left represents CD44+ 1807 CD4+ cells in infected WT mice, amounting to just over 10E3. Where is the bar for naïve WT mice? In Fig 1A it shows this should be up to over 10E2. That would be higher than in all the different naïve KO mice. Where are those WT responses coming from?

Fig 3B why no frequencies?

The legend of Fig 3. statistical significance is mentioned indicated with *. But none of the differences are significant. Still the authors conclude on p7 that -/- mice for Dectin-1,-2 , Card9, CLEC4 have reduced activated cells. I don't think this can be concluded from Fig 3.

It may be better to leave out Fig 3 altogether as it does not seem to contribute to the paper.

The authors mention that endogenous T-cells follow the trend for 1807 cell, but – logically – alterations for the antigen-specific 1807 cells are larger and provide better resolution. In 4C and D the two are indicated next to each other, and the relative numbers indeed move in the same direction. But, except for Card9, in those cases where statistical significance is observed either the endogenous cell differentiation is significantly altered or the 1807 cell differentiation, but never both. It all looks very borderline significant to me, suggesting it could be questioned whether it would be allowed to draw the specific conclusions about Dectin -2 and Mincle.

<u>First revision – authors' response – 6 May 2015</u>

REVIEWER 1

1. Query: Using a subcutaneous infection model of F. pedrosoi, the authors observed increased activation (as determined by CD44+ expression) and effector differentiation (IFN-g and IL-17 production) of endogenous and 1807 TCR-transgenic CD4+ T cells in response to infection. Adoptively transferred 1807

European Journal of Immunology

T cells appear uniformly CD44+ already prior to infection (Fig. 1C), raising the question whether the increase in cell number upon infection reflects bona fide T cell activation or rather expansion of pre-activated/memory T cells, given that adoptively transferred cells were not restricted to naïve (CD44-, CD62L+) cells. Monitoring T cell proliferation by CFSE-dilution may thus provide a more sensitive readout for the T cell responsiveness in the different conditions. Also, in Fig. 3B, the CD44+ population within the Thy1.1 TCR-tg cells is gated differently for the naïve and the infected samples, which greatly biases the results.

Response: The reviewer points out correctly that some adoptively transferred 1807 T cells were pre-activated (upon re-examination about 50-60% were CD44 positive in the top left panel in Fig. 1C). To address the concern, we repeated the experiment (twice) and determined the frequency of CD44high cells among the donor 1807 cells (<10%) (data not shown) prior to transfer. Using CD44low donor 1807 cells (>90%), the results were similar to the original data. Mice infected with F. pedrosoi spores showed strong activation and expansion of fungus-specific 1807 cells. Co-stimulation with LPS or Imiquimod did not lead to augmented numbers (Fig. 1A-C) and frequencies of activated and cytokine producing (Fig. 1D+E) 1807 cells. We have replaced the original data with new data from a representative repeat experiment and also generated the new panels D+E in response to reviewer 3. We report these new data in the results section on page 6, 1st para, lines 6-8.

With regard to the gating on CD44-positive 1807 cells in Fig. 3, we have revised the figure as follows. We have increased the threshold for CD44 positive 1807 cells in infected mice in Fig. 3C. However, the threshold for CD44 expression by 1807 cell vs. endogenous CD44+ T cell needs to be set lower. We previously observed that vaccine antigen dose influences the expression of CD44 on CD4+ TCR transgenic T cells (Infect Immun 2012; 80:787-97); specifically, an increasing antigen dose is associated with diminished intensity of expression (not frequency) on CD4+ T cells. For this reason, the dot plots in Fig. 3C required that we employ different gates. We believe that we have set the gates reasonably and that the CD44hi cells segregate themselves appreciably among the different groups.

We have also generated a new panel 3B (according to the suggestion of reviewer 3), in which we show the percentage of CD44+ 1807 cells in naïve and infected mice. Less than 10% of transferred 1807 cells express CD44 in naïve mice, whereas 40-70% 1807 cells are activated in infected mice. These data show that >90% 1807 cells in naïve mice are CD44 negative, which then convert to CD44 positive T cells after infection. To better illustrate the number of adoptively transferred 1807 cells in naïve strains of recipient mice we enumerated the total number of 1807 cells rather than CD44 positive cells (Fig. 3C).

2. Query: Cytokine production was assessed after polyclonal re-stimulation, which leaves open the question to what extent the endogenous response is actually specific for the fungus.

European Journal of Immunology

Response:
Throughout the paper we focus on and mainly discuss the data generated with fungus-specific 1807 cells. Thus, ex vivo stimulation with anti-CD3 and CD28 of 1807 cells is reporting cytokine production by fungus-specific T cells. With regard to endogenous CD4 T cells, the reviewer is correct. “Polyclonal stimulation” could engage cytokine production by non-fungus specific T cells as well. However, by and large, the endogenous CD4+ T cell responses correlated with 1807 responses although they do not provide the same degree of resolution as the reviewer points out correctly. We acknowledge that fact on page 5, last paragraph.

3. Query: In Fig. 1, co-stimulation of F. pedrosoi-infected mice with LPS did not lead to enhanced T cells responses. If anything, it led to a reduction of the antifungal response, something the authors did not comment on. The question arises whether the timing of administering the LPS was ideal. Also, the authors should provide a control for the activity of the LPS (e.g. measuring TNF in the serum).

Response: To investigate whether LPS stimulation of F. pedrosoi-infected mice reduces T cell responses, we repeated the experiment twice (as described in Query 1). The number of activated (CD44+) and cytokine producing 1807 T cells did not change significantly in either experiment. In addition, similar results were generated with Imiquimod (see revised Fig. 1).

We also injected naïve mice with 100ng LPS i.p. (which is the concentration and route that we used for TLR co-stimulation in F. pedrosoi-infected mice) and measured TNF-α transcript in PBMC. LPS injected mice showed a twenty-fold increase in transcript vs. naïve mice. Mice injected with 5 µg of LPS did not show augmented transcript levels vs. 100 ng LPS. These data indicate that the concentration, formulation and mode of delivery of LPS used was bio-active. We provide that information on page 13, last para, second to last sentence.

4. Query: The authors show that Th17 induction in response to F. pedrosoi is not reduced in Mincle-/- mice (Fig. 3 and 4) and that F. pedrosoi does not bind to Mincle, but to Dectin-2 (Fig. 2). These data are conflicting with previous findings from the authors, which showed that absence of Mincle but not Dectin-2 signaling led to a reduced inflammatory response to F. pedrosoi (de Gloria Sousa, Cell Host and Microbe, 2011). To what extent are the differences between the two reports explained by differences in the experimental systems used? While in this study they used a BWZ assay to assess binding of F. Pedrosoi to Mincle (and Dectin-2), they analyzed DCs in their previous study for their capacity to produce cytokines in absence of Mincle (or Dectin-2) signaling. Because T cell differentiation depends on APC-derived cytokines, cytokine production by APCs in response to F. pedrosoi is an important parameter for explaining the observed effects in Figure 3 and 4. Therefore, to support their current data (and possibly to clarify discrepancies arising from the two studies), the authors should look at relevant APCs in the infected

European Journal of Immunology

organ and/or in the draining LN of infected mice and compare their cytokine profile in WT, Mincle-/- and Dectin-2-/- mice.

Response: In the CHM paper cited, we measured in vitro TNF production by macrophages and DC as the main outcome to investigate TLR costimulation. However, TNF is not a priming cytokine for Th17 and Th1 cells, nor does it induce proliferation of T cells. In this manuscript we measured F. pedrosoi-specific T cell responses and found that T17 cell differentiation is altered in Mincle-/- and Dectin-2-/- mice as shown in Fig. 4. Thus, we believe the data in these two papers are not conflicting because they show distinct outcomes that are not related. We would also point out that we did show low level recognition of live spores by Mincle-expressing reporter cells (in Fig. 2), but this might reflect receptor expression level or lack of complexity of pathogen recognition by the reporter cells.

5. Query: Linked to the above, the authors should check for fungal load in Mincle-/- mice in their model. If Mincle-/- mice have a defective innate control and thus increased burden of F. pedrosoi (as described by de Gloria Sousa et al, Cell Host and Microbe, 2011), this may affect the extent of the adaptive response. Increased fungal load (and thus increased availability of antigen) may lead to an enhanced adaptive response, and the contribution of Mincle to T cell priming may thereby be masked. Similarly, the effect of Card9 and FcRg on T cell priming in response to F. pedrosoi may be underestimated. Similarly, I wonder to what extent the increased Th1 response in MyD88-/- mice impacts on the reduced Th17 response in these mice, while the increased Th17 response in Mincle-/- may affect the Th1 subset (or vice versa).

Response: The reviewer correctly points out that we have not enumerated the fungal burden in the various knockout and wild type recipients of adoptively transferred 1807 cells. It is possible that Mincle-/- mice (the fungal burden was not measured in the CH&M article) and Dectin-2-/- mice have a different fungal burden than wild type mice as reported for Dectin-1-/- and Myd88-/- mice in CH&M, Brown et al.. According to the reviewer's suggestion, on page 11, last para, lines 3 to 7, we acknowledge that a difference in the fungal burden may have impacted adaptive T cell responses in Mincle-/- vs. wild type mice. Nevertheless, independent of any differences in the fungal burden we found no alteration in the activation and expansion (Fig. 3), whereas differentiation (Fig. 4) of adoptively transferred 1807 cells was altered.

6. Query: Th17 induction is incomplete in both, Card9-/- and MyD88-/-, which suggest that additional innate pathways may contribute, or that the Dectin-2/Card9 and the MyD88 pathways are partially redundant. This could be tested experimentally e.g. by generating double-deficient mice or by blocking Dectin-2 in MyD88-/- mice.

Response: The reviewer makes a good point. It is possible that the Myd88 and Card9 signaling axis are partially redundant for the development of Th17 cells. Unfortunately, it would take us several months to

European Journal of Immunology

cross Myd88-/- and Card9-/- mice and anti-Dectin-2 blocking antibodies are not commercially available. Thus we consider the suggested experiments outside the scope of this manuscript.

7. Query: How do the authors conciliate their finding that 1807 T cells were reduced in Decint-2-/-, but not FcRg-/- mice (Fig. 3) with the essential role of FcRg for Dectin-2 surface expression?

Response: Differences in the number of activated 1807 T cells in Dectin-2-/-, FcRγ-/- and WT mice in Fig. 3 are not significant as acknowledged in the text of the manuscript on page 7, 2nd para, lines 5-7. However, we show that the frequency and number of IL-17 producing 1807 T cells is reduced in Dectin-2-/- vs. WT mice (Fig. 4A-C). From these data, we concluded that the Dectin-2/FcRγ/Card9 signaling axis is required for Th17 cell differentiation.

Minor points:
The sentence 'Dectin-2 mediated recognition of F. pedrosoi spores is REQUIRED for development of Ag-specific Th17 cells' (page 9, 1st paragraph) is overstated and should be changed. There is only a partial reduction of Th17 induction observed in Dectin-2-deficient mice (Fig. 4).

Response: The reviewer is correct that Dectin-2 is not solely responsible for the induction of Th17 cell differentiation. We have changed the statement to read: ”Dectin-2 (and to a lesser extent Dectin-1) mediated recognition of F. pedrosoi spores is largely responsible for the development of Ag-specific Th17 cells. This modification can be found on p. 9, 1st para, line 5 of the revision.

On page 10, towards the end of the 1st paragraph, the authors say 'Thus, increased Mincle expression… in the reporter assay in which Mincle and MCL were NOT co-expressed.', but they actually included a condition of Mincle and MCL coexpression in Fig. 2.

Response: The reviewer is correct. We eliminated that statement.

On page 10, 2nd paragraph, reference 21 is not appropriate for 'IL-17 responses in antifungal immunity in models of systemic candidiasis (ref. 21 does not show any data on systemic candidiasis).

Response: The review is correct. We have added two references that include literature on systemic candidiasis. This modification can be found on p. 10, last para, line 20 of the revision.

REVIEWER 2

Comments to the Author

European Journal of Immunology

This study describes investigates the role of C-type lectin receptors (CLR) in adaptive immune responses to F. pedrosoi. Previously, the authors have shown that TLR co-stimulation is required to induce innate immunity to F. pedrosoi, since Mincle activation alone is not sufficient for host defence. Here the authors show that TLR-costimulation does not affect adaptive immunity as assessed by Ag-specific Th17 activation. Using CLR deficient mice, they show that the immune responses (Th17) are severely impaired in dectin-1 and dectin-2 deficient mice, whereas Mincle deficient mice have higher Th17 responses. The authors conclude that dectin-1 and -2 are required for adaptive immunity to F. pedrosoi whereas Mincle seems to suppress Th17 responses.

1. Query: The lack of TLR co-stimulation is striking since the authors have previously shown that co-stimulation is required for induction of innate responses. These data suggest that F. pedrosoi can induce cytokines via dectin-1 and -2, in contrast to the previous study. In vitro stimulation of DC from different CLR deficient mice would support the suggested roles for the CLR in infections especially the role for MCL and Mincle.

Response: Our initial CHM publication does not demonstrate a role for Dectin-1 in F. pedrosoi recognition, but not in costimulatory responses. However, to address the reviewers concern we conducted the coculture experiments with BMDC and spores as the reviewer suggests. We chose to measure IL-6, a critical priming cytokine required for the differentiation of Th17 cells. Compatible with our in vivo results, Dectin-1-, Dectin-2-, FcRγ-, Card9- and Myd88-deficient DC produced significantly less IL-6 than wild-type DC (data not shown). However, Mincle-/- DC did not produce higher amounts of IL-6 as one would expect from the in vivo experiments in our current paper. We believe that the in vitro assay with BMDC is a reductionist approach, which may not accurately model the in vivo events leading to adaptive responses. For example, BMDC are in many ways different from regional antigen presenting cells (APC) that present fungal Ag in vivo. Cytokine(s) that prime T cells might be a result of interactions of multiple subsets of APC and cytokines other than IL-6 could influence differentiation of Th17 cells etc. For these reasons, we chose not to include these in vitro data into the manuscript.

Major concerns

2. Query: Figure 1. The use of fungus-specific T cells is interesting but it is not clear whether the induction of the 1807 T cells is fungus specific. Controls should be included to demonstrate that the increased proliferation is not due to general inflammation. The induction of endogenous T cells suggests non-specific activation.

Response: We previously demonstrated that 1807 T cells are activated only by fungi that contain the corresponding calnexin epitope, but not all fungi (Fig. 1 in Journal of Immunology 2011, 187:1421 and Fig. 3 in Cell Host and Microbe 2015, 17:1-14). For example, vaccination or infection with Candida albicans,

European Journal of Immunology

Cryptococcus neoformans and Pnemocystis carinii do not trigger the activation and proliferation of naïve 1807 T cells, whereas many ascomycete fungi do trigger these T cells.

3. Query: Figure 2. F. pedrosoi has been shown to bind Mincle but using the cell-line, Mincle and MCL activation are rather small, even though the authors suggest that Mincle is important in suppressing Th17 responses. The small increase in activation does not explain the strong effect on Th17 responses. This should be further investigated. In vitro stimulations using Mincle deficient mice would provide a more clear answer. This is important since previous studies using mycobacterial ligand strongly suggest that Mincle enhances/induces Th17 responses.

Response: The reviewer is correct, increased Th17 responses in Mincle-/- mice could not be explained by the reporter cell data. As suggested by the reviewer, we conducted additional in vitro experiments to investigate the discrepancy. First, in addition to live spores, we sonicated or lysed spores and tested the supernatants of the sonicate and lysates in the reporter assay to investigate whether Mincle recognizes a cell wall, membrane or cytosolic ligand from F. pedrosoi spores. Supernatants after sonication and lysates trigger strong reporter activity with Dectin-1 and Dectin-2, but not with Mincle-expressing cell lines.

Second, we co-cultured Mincle-/- BMDC with F. pedrosoi spores and measured IL-6 in the cell culture supernatant by ELISA. Mincle-/- DC did not show enhanced IL-6 production compared to wild type DC as expected and suggested by the reviewer (data not shown). Please also see response to Query 1. We conclude that these two in vitro assays are reductionist approaches that do not fully recapitulate the in vivo setting and observation. It is conceivable that Mincle is part of a receptor complex that regulates Th17 responses, thus the reporter assay does not reflect this complexity. In addition, the receptor complex could be present in antigen presenting cells in the popliteal lymph node that stimulate the T cells, but not in the bone marrow derived dendritic cells that we used in vitro. We now discuss this point in the discussion on page 11, last para, lines 20-23.

We agree with the reviewer that Mincle can function both as an activating and inhibiting receptor as discussed on page 11, last para and page 12 1st para. While there is a larger body of literature describing Mincle as an activating receptor, Mincle has also been reported to suppress Th1 responses to Fonsecaea monorpha (Cell Host and Microbe 2014, 15: 494-505).

4. Query: Figure 3. the T cells in naïve mice are already different in the CLR deficient mice. This could impact the observed proliferation during infection. How did the authors control for this? Mincle and Clec4d deficient mice have already higher 1807 T cell-numbers in naïve mice. Could this affect the observed Th17 responses observed in Figure 4. In vitro stimulations are required to support the hypothesis of the authors concerning dectin-1, -2 and mincle in infection.

European Journal of Immunology

Response: The reviewer is correct, portraying the number of activated (CD44+) 1807 cells in naïve mice in Fig. 3 creates the impression that the number of 1807 cell precursors is different in Mincle-/- vs. corresponding wild type controls. Since less than 10% of transferred 1807 cells are activated (CD44+)(as shown in newly generated Fig. 3B), we chose to illustrate total (CD44low and CD44high) number of 1807 cells in naïve mice instead (Fig. 3A+C). The total number of 1807 cells in naïve Mincle-/- vs. wild type mice is comparable (Fig. 3A+C). In summary, the adoptively transferred 1807 cells were mostly CD44low and input frequencies of 1807 cells were comparable between knockout and wild type controls. Thus, we conclude that the increased Th17 responses in Mincle-/- vs. wild type mice is likely not due to differences in the T cell precursor input.

As discussed in response to Query 1, we measured IL-6 production by F. pedrosoi-stimulated BMDC. We have been able to confirm the in vivo phenotype with regard to Dectin-1 and Dectin-2, but not Mincle. We strongly believe that the proposed in vitro assay is a reductionist approach to investigate the role of these CLR in inducing Th17 responses and does not accurately represent in vivo events. For these reasons we chose not to show the in vitro results since they will likely be more confusing than helpful.

REVIEWER 3

Comments to the Author
This manuscript primarily addressed the question if co-stimulation by TLR agonists fosters the development innate and also adaptive immune responses to F pedrosoi, by promoting development of T-cells specific to the fungus. While Fp Ag specific CD4+ T-cells are induced upon infection, from the initial set of experiments it seems that TLR agonists do not promote the T-cell development further. The authors proceeded to carry out basic experiments to investigate which C-type lectins, some of which had been implicated in the innate responses to F pedrosoi, may be involved in augmenting T-cell responses. It is concluded that among a set of lectins possible involved in recognition of Fp antigens, Dectin-2 via the FcRg/Card9 pathway promotes differentiations of CD4+ into Th17 cells whereas Mincle engagement inhibits.

In general this manuscript contains interesting information, as far as I am aware as a non-specialist in fungal infection immunology, but the way the data are presented now and the questions the authors attempt to answer are not always clear to me. To the primary question - do TLR agonists stimulate/augment fungus specific T cell development - the answer quite clearly no. But the rest of paper becomes less clear as it twists in a slightly different direction (with some questions about the data below), with a more preliminary and open end.

Specific questions/comments:

European Journal of Immunology

1. Query: Fig 1. The statistical relevance indication as mentioned in the legend is lacking in the figure. Why are frequencies not given, as in Fig 4C, this would be helpful to see what is going on more easy.

Response: TLR costimulation did not augment T cell activation, expansion and differentiation. Thus, the data are not statistically different. We have now clarified the results of the statistical analysis in the figure legend. As suggested by the reviewer we also generated a new dot plot (Fig. 1D) and histogram (Fig. 1E) showing the frequencies of cytokine producing and CD44 positive 1807 cells.

2. Query: In relation to figure 2, except for Mincle, which was implied in Fp immunity previously, what is the rationale of investigating this specific set of C-type lectin signaling?

Response: It is now widely accepted that fungi are principally recognized by the TLR/Myd88 and CLR/Card9 signaling pathways (Annual Review in Immunology 2012, 24: 1-10). All the C type lectin receptors investigated in this manuscript have previously been shown to recognize fungal PAMPs.

3. Query: From Fig 2. is was concluded that Dectin-1 and -2 induce strong signalling and Mincle only weak. Mincle is very weak in fact, surprising in view of the later conclusions about Mincle involvement in Fp immunity. Or is it possible that Dectin is more highly expressed on the cells, hence gives a higher signal? A positive control for each lectin would have been necessary to draw conclusions about relative strong or weak signalling.

Response: We stimulated each reporter cell line with a corresponding positive control to verify functionality (data not shown). The positive controls were as follows: H. capsulatum yeast for Dectin-1 and B. dermatitidis for Dectin-2 (J Immunol 2014, 192(3): 1107), and TDB for Mincle and MCL.

Constitutive expression of a single (or in some cases co-expression of two) CLR on the surface of a T cell hybridoma may not match in vivo receptor expression by APCs. It is also conceivable that multiple receptors form a receptor complex in vivo to facilitate receptor collaboration, which is not reflected with the reporter cells. For these reasons, signal strength by reporter cells and in vivo relevance of the CLR may not always completely match (e.g. in the case of Mincle). However, in the case of Dectin-1, Dectin-2, and MCL the reporter assay forecasted in vivo relevance well.

4. Query: Fig 3A. The bar in the left represents CD44+ 1807 CD4+ cells in infected WT mice, amounting to just over 10E3. Where is the bar for naïve WT mice? In Fig 1A it shows this should be up to over 10E2. That would be higher than in all the different naïve KO mice. Where are those WT responses coming from?

European Journal of Immunology

Response: The number of activated (CD44+) 1807 cells in naïve wild type mice, as shown in Fig. 3C, is "one". We realize that illustrating number of activated (CD44+) 1807 cells in naïve mice can be confusing. Thus, we decided to enumerate and illustrate total number of transferred 1807 cells, which are the precursors for F. pedrosoi induced T cell activation and expansion. Please also see our response to reviewer 2, query 4. The number of total 1807 cells in knockout vs. wild type recipient mice is largely comparable and yields about a ten-fold expansion in the number of activated 1807 cells (Fig. 3A+C).

5. Query: Fig 3B why no frequencies?

Response: As requested by the reviewer, we now show the frequencies of CD44+ 1807 cells in Panel B of Fig. 3. There were no statistically significant differences between knockout vs. wild type controls.

6. Query: The legend of Fig 3. statistical significance is mentioned indicated with *. But none of the differences are significant. Still the authors conclude on p7 that -/- mice for Dectin-1,-2 , Card9, CLEC4 have reduced activated cells. I don't think this can be concluded from Fig 3.

Response: Although there is a trend, the numbers of CD44+ 1807 T cells are not significantly reduced between Card9-/-, Dectin-1-/- and Dectin-2-/- vs. wild-type mice. We have highlighted the corresponding sentence on page 7, 2nd para and now state it in the legend of Fig. 3 too.

7. Query: It may be better to leave out Fig 3 altogether as it does not seem to contribute to the paper.

Response: We would like to keep Fig. 3 in the manuscript for the following reason. We developed and successfully used adoptive transfer of 1807 cells because it allows us to carefully pinpoint at what stage of the immune responses T cell extrinsic factors influence their development (see Journal of Immunology 2014, 192(3): 1107-19). Here, the adoptive transfer of 1807 cells indicated that T cell differentiation, but not activation and expansion, is affected by the Dectin-2/FcRγ/Card9 signaling axis. While Fig. 3 shows no phenotype by the CLR and adaptor molecules investigated, it is an integral part of our stage-by-stage analysis of T cell development.

8. Query: The authors mention that endogenous T-cells follow the trend for 1807 cell, but – logically – alterations for the antigen-specific 1807 cells are larger and provide better resolution. In 4C and D the two are indicated next to each other, and the relative numbers indeed move in the same direction. But, except for Card9, in those cases where statistical significance is observed either the endogenous cell differentiation is significantly altered or the 1807 cell differentiation, but never both. It all looks very borderline significant to me, suggesting it could be questioned whether it would be allowed to draw the specific conclusions about Dectin -2 and Mincle.

Response: The reviewer points out correctly that 1807 T cells provide better resolution since they are all fungus-specific, whereas this is not the case for endogenous CD4+ T cells. Since Card9 funnels signaling of multiple CLR it is not surprising that Card9-/- mice showed a stronger phenotype than mice lacking a single CLR. Nevertheless, the number of IL-17 producing 1807 (and endogenous CD4+) T cells is significantly reduced in Dectin-2-/- vs. wild type mice (Fig. 4A+B). Likewise, the frequency of IL-17+ 1807 cells is significantly increased in Mincle-/- mice. Although not statistically significant, Mincle-/- mice show a trend towards increased Th17 cell numbers. In addition, we believe that a trend towards a phenotype can be biologically relevant without being statistically significant.

Second Editorial Decision – 3 June 2015

Dear Dr. Wuethrich,

Thank you for submitting your revised manuscript ID eji.201545591.R1 entitled "Engagement of C-type lectin receptors by Fonsecaea pedrosoi spores induces differential T cell immunity" to the European Journal of Immunology. Your manuscript has been re-reviewed and the comments of the referees are included at the bottom of this letter.

Although the referees have recommended publication, some revisions to your manuscript have been requested. Therefore, I invite you to respond to the comments of the referees and revise your manuscript accordingly.

You should also pay close attention to the editorial comments included below. *In particular, please edit your figure legends to follow Journal standards as outlined in the editorial comments. Failure to do this will result in delays in the re-review process.*

If the revision of the paper is expected to take more than three months, please inform the editorial office. Revisions taking longer than six months may be assessed by new referees to ensure the relevance and timeliness of the data.

Once again, thank you for submitting your manuscript to European Journal of Immunology. We look forward to receiving your revision.

Yours sincerely,
Karen Chu

on behalf of Prof. Maria Yazdanbakhsh

Peer review correspondence

Dr. Karen Chu
Editorial Office
European Journal of Immunology
e-mail: ejied@wiley.com
www.eji-journal.eu

****

Reviewer: 1

Comments to the Author
Wüthrich et al. have addressed most of my concerns in the revised version of their manuscript. The additions made to the figures and the text strengthen the quality of the manuscript significantly. I only have two comments:

- In Fig. 3 the authors changed the gate for the CD44+ 1807 Tg CD4+ T cells. However, for the infected samples they did not adjust the number of cells within the gates (Fig. 3C, and possibly 3A-B), while the numbers were adjusted in case of the naïve samples.

- The authors mention in their responses to the reviewers that they stimulated BMDCs from the different KO mice with F. pedrosoi spores and analyzed the induction of IL-6, a cytokine involved in Th17 differentiation. These data should be included in the manuscript (e.g. as a Figure 2b) for the following reasons:
The induction of IL-6 by F. pedrosi seems to be consistent with the induction of the Th17 response, which are both reduced in absence of Dectin-2, but not in absence of Mincle. Although the response of BMDCs in vitro does indeed not fully reflect the complexity of the in vivo situation at the site of infection/in the draining lymph node, the data do provide a certain link, which was so far missing, between the data shown in Fig. 2 (binding of F. pedrosi spores to Dectin-2 but not Mincle in the BWZ assay) and the data shown in Fig. 4 (induction of Th17 differentiation). Furthermore, the data illustrate that different responses to F. pedrosi are regulated differentially via different CLRs (TNF production vs. Th17 differentiation). They thus provide at least a partial explanation for the differences in Mincle- and Dectin-2 depencence observed between this and their previous study (CHM 2011) and provide a basis for further investigations.
Although IL-6 production by BMDCs does not reflect all aspects of Th17 differentiation by different CLRs in vivo (e.g. no enhanced IL-6 production by Mincle-/- BMDCs), the data do add to the manuscript and should thus be included.

European Journal of Immunology

Reviewer: 2

Comments to the Author
The authors have addressed the concerns raised but the lack of in vitro proof for Mincle function is a concern (Q1,3 and 4). The authors should clearly state this in the discussion. it is possible that Mincle deficient mice have other defects which allow for the increased Th17 responses or that these responses are secondary? Furthermore, did the authors investigate more than only the IL-6 responses by Mincle-deficient BMDC?

Reviewer: 3

Comments to the Author
The authors have answered my questions satisfactorily. The made quite extensive adaptations and improvements to the mansucript. The data still do not always allow clear conclusions, or conclusions depend on the interpretations. Overall the revision improved the paper significantly in my opinion.

Second revision – authors' response – 8 June 2015

REVIEWER 1

Comments to the Author
Wüthrich et al. have addressed most of my concerns in the revised version of their manuscript. The additions made to the figures and the text strengthen the quality of the manuscript significantly. I only have two comments:

Query 1: In Fig. 3 the authors changed the gate for the CD44+ 1807 Tg CD4+ T cells. However, for the infected samples they did not adjust the number of cells within the gates (Fig. 3C, and possibly 3A-B), while the numbers were adjusted in case of the naïve samples.

Response: The reviewer noticed correctly that the number of activated (CD44+) T cells of infected mice did not change in the dot plots in Fig. 3C even though we have tightened the gates in the revised figure. This is because we determined the number of activated Thy1.1+ (transgenic) and endogenous CD4+ T cells by gating on these two populations separately, even though we display them together in a Thy1.1+ vs. CD44+ dot plot for ease of comparison. This allowed us to draw the threshold for CD44+ cells more precisely for enumeration than what we show in concatenated samples in Fig. 3C where we display endogenous and transferred CD4+ T cells together for the sake of a concise presentation.

European Journal of Immunology

Query 2: The authors mention in their responses to the reviewers that they stimulated BMDCs from the different KO mice with F. pedrosoi spores and analyzed the induction of IL-6, a cytokine involved in Th17 differentiation. These data should be included in the manuscript (e.g. as a Figure 2b) for the following reasons:
The induction of IL-6 by F. pedrosi seems to be consistent with the induction of the Th17 response, which are both reduced in absence of Dectin-2, but not in absence of Mincle. Although the response of BMDCs in vitro does indeed not fully reflect the complexity of the in vivo situation at the site of infection/in the draining lymph node, the data do provide a certain link, which was so far missing, between the data shown in Fig. 2 (binding of F. pedrosi spores to Dectin-2 but not Mincle in the BWZ assay) and the data shown in Fig. 4 (induction of Th17 differentiation). Furthermore, the data illustrate that different responses to F. pedrosi are regulated differentially via different CLRs (TNF production vs. Th17 differentiation). They thus provide at least a partial explanation for the differences in Mincle- and Dectin-2 depencence observed between this and their previous study (CHM 2011) and provide a basis for further investigations.
Although IL-6 production by BMDCs does not reflect all aspects of Th17 differentiation by different CLRs in vivo (e.g. no enhanced IL-6 production by Mincle-/- BMDCs), the data do add to the manuscript and should thus be included.

Response: Based on the reviewer's suggestion, we have incorporated the IL-6 data into Fig. 2B. We report the data in the results section on page 8, 2nd para and discuss them in the discussion on page 11, 1st para, lines 7-10 and 2nd para, lines 22-24 and page 12, 1st para, lines 1-3.

REVIEWER 2

Comments to the Author
The authors have addressed the concerns raised but the lack of in vitro proof for Mincle function is a concern (Q1,3 and 4). The authors should clearly state this in the discussion. It is possible that Mincle deficient mice have other defects which allow for the increased Th17 responses or that these responses are secondary? Furthermore, did the authors investigate more than only the IL-6 responses by Mincle-deficient BMDC?

Response: We agree with the reviewer. Thus, on page 11, 2nd para, lines 21-23 (underlined) we state explicitly that the Th17 responses regulated by Mincle in vivo were not reflected by the lack of reporter activity by Mincle-expressing reporter cells, nor by the increased IL-6 production by Mincle-/- BMDCs. We did not measure any other cytokine produced by spore-stimulated BMDC because we felt that IL-6 is one of the key cytokines that drives Th17 cell differentiation. However, the reviewer is correct, other in vitro produced priming cytokines might have better supported the in vivo phenotype in the Mincle-/- mice. We acknowledge this possibility in the discussion on page 11, last sentence and on page 12, first sentence. In

European Journal of Immunology

addition, we have also previously acknowledged that the Mincle Th17 cell phenotype could be secondary to increased the fungal burden, as stated on page 11, 2nd para, lines 17-19 (underlined sentence).

REVIEWER 3

Comments to the Author
The authors have answered my questions satisfactorily. The made quite extensive adaptations and improvements to the manuscript. The data still do not always allow clear conclusions, or conclusions depend on the interpretations. Overall the revision improved the paper significantly in my opinion.

Response: We are pleased that this reviewer is satisfied with our revised manuscript.

Third Editorial Decision – 12 June 2015

Dear Dr. Wuethrich,

It is a pleasure to provisionally accept your manuscript entitled "F. pedrosoi-induced Th17-cell differentiation in mice is fostered by Dectin-2 and suppressed by Mincle recognition" for publication in the European Journal of Immunology. For final acceptance, please follow the instructions below and return the requested items as soon as possible as we cannot process your manuscript further until all items listed below are dealt with.

Please note that EJI articles are now published online a few days after final acceptance (see Accepted Articles: http://onlinelibrary.wiley.com/journal/10.1002/(ISSN)1521-4141/accepted). The files used for the Accepted Articles are the final files and information supplied by you in Manuscript Central. You should therefore check that all the information (including author names) is correct as changes will NOT be permitted until the proofs stage.

We look forward to hearing from you and thank you for submitting your manuscript to the European Journal of Immunology.

Yours sincerely,
Karen Chu

on behalf of Prof. Maria Yazdanbakhsh

Peer review correspondence

Dr. Karen Chu
Editorial Office
European Journal of Immunology
e-mail: ejied@wiley.com
www.eji-journal.eu
